# Sex- and age-related differences in the inflammatory properties of cardiac fibroblasts: impact on the cardiosplenic axis and cardiac fibrosis

**DOI:** 10.3389/fcvm.2023.1117419

**Published:** 2023-11-20

**Authors:** Kathleen Pappritz, Sarah-Lena Puhl, Isabel Matz, Erik Brauer, Yi Xuan Shia, Muhammad El-Shafeey, Suzanne E. Koch, Kapka Miteva, Christin Mucha, Georg N. Duda, Ansgar Petersen, Sabine Steffens, Carsten Tschöpe, Sophie Van Linthout

**Affiliations:** ^1^ Berlin Institute of Health at Charité—Universitätsmedizin Berlin, BIH Center for Regenerative Therapies (BCRT), Berlin, Germany; ^2^ Berlin-Brandenburg Center for Regenerative Therapies, Charité—Universitätsmedizin Berlin, Campus Virchow Klinikum (CVK), Berlin, Germany; ^3^ German Center for Cardiovascular Research (DZHK), Partner Site Berlin, Berlin, Germany; ^4^ Comprehensive Heart Failure Center, Universitätsklinikum Würzburg, Würzburg, Germany; ^5^ Institute for Cardiovascular Prevention (IPEK), Ludwig-Maximilians-Universität (LMU) Munich, Munich, Germany; ^6^ Berlin Institute of Health at Charité—Universitätsmedizin Berlin, Julius Wolff Institute, Berlin, Germany; ^7^ Medical Biotechnology Research Department, Genetic Engineering and Biotechnology Research Institute (GEBRI), City of Scientific Research and Technological Applications, Alexandria, Egypt; ^8^ Department of Biomedical Engineering, Eindhoven University of Technology, Eindhoven, Netherlands; ^9^ Division of Cardiology, Foundation for Medical Research, Department of Medicine Specialized Medicine, Faculty of Medicine, University of Geneva, Geneva, Switzerland; ^10^ German Center for Cardiovascular Research (DZHK), Partner Site Munich Heart Alliance (MHA), Munich, Germany; ^11^ Department Cardiology, Angiology, and Intensive Medicine (CVK) at the German Heart Center of the Charite (DHZC), Charité—Universitätsmedizin Berlin, Berlin, Germany; ^12^ Institute of Heart Diseases, Wroclaw Medical University, Wroclaw, Poland

**Keywords:** aging, sex, cardiac fibroblasts, cardiosplenic axis, monocytes, fibrosis

## Abstract

**Background:**

Age and sex are prominent risk factors for heart failure and determinants of structural and functional changes of the heart. Cardiac fibroblasts (cFB) are beyond their task as extracellular matrix-producing cells further recognized as inflammation-supporting cells. The present study aimed to evaluate the impact of sex and age on the inflammatory potential of cFB and its impact on the cardiosplenic axis and cardiac fibrosis.

**Materials:**

Left ventricles (LV) of 3- and 12-months old male and female C57BL/6J mice were harvested for immunohistochemistry, immunofluorescence and cFB outgrowth culture and the spleen for flow cytometry. LV-derived cFB and respective supernatants were characterized.

**Results:**

LV-derived cFB from 3-months old male mice exhibited a higher inflammatory capacity, as indicated by a higher gene expression of CC-chemokine ligand (CCL) 2, and CCL7 compared to cFB derived from 3-months old female mice. The resulting higher CCL2/chemokine C-X3-C motif ligand (Cx3CL1) and CCL7/Cx3CL1 protein ratio in cell culture supernatants of 3-months old male vs. female cFB was reflected by a higher migration of Ly6C^high^ monocytes towards supernatant from 3-months old male vs. female cFB. *In vivo* a lower ratio of splenic pro-inflammatory Ly6C^high^ to anti-inflammatory Ly6C^low^ monocytes was found in 3-months old male vs. female mice, suggesting a higher attraction of Ly6C^high^ compared to Ly6C^low^ monocytes towards the heart in male vs. female mice. In agreement, the percentage of pro-inflammatory CD68^+^ CD206^−^ macrophages was higher in the LV of male vs. female mice at this age, whereas the percentage of anti-inflammatory CD68^+^ CD206^+^ macrophages was higher in the LV of 3-months old female mice compared to age-matched male animals. In parallel, the percentage of splenic TGF-*β*^+^ cells was higher in both 3- and 12-months old female vs. male mice, as further reflected by the higher pro-fibrotic potential of female vs. male splenocytes at both ages. In addition, female mice displayed a higher total LV collagen content compared to age-matched male mice, whereby collagen content of female cFB was higher compared to male cFB at the age of 12-months.

**Conclusion:**

Age- and sex-dependent differences in cardiac fibrosis and inflammation are related to age- and sex-dependent variations in the inflammatory properties of cardiac fibroblasts.

## 1. Introduction

Aging is an important risk factor for developing cardiovascular diseases (CVD) (1, 2), including heart failure (HF) (3). Due to improved health care, lifespan is constantly increasing and in 2030, it is estimated that around 20% of the population worldwide will be over 65 years (4). Within this age-group, CVD will cause 40% of all deaths (4). Although it is known that in addition to age, also sex influences cardiac function (5), little attention has been devoted to its role in HF pathology. Intriguingly, before the age of 65, more men than women die from CVD, whereas beyond this age death rates of women increase (6), probably explained by declining estrogen levels (5). These sex differences affect myocardial performance at functional (7), structural (8, 9) as well as cellular level (10, 11). It is well accepted that during the progression of HF, the immune system is locally and systemically activated followed by a broad interaction with the cellular components of the heart (12, 13).

In detail, cFB respond to growth factors, cytokines, and chemokines, followed by a phenotypic switch to myofibroblasts (14, 15), resulting in an increased collagen/extracellular matrix (ECM) deposition. Furthermore, cFB can self-maintain the inflammatory and fibrotic processes in the heart via production and secretion of pro-inflammatory chemokines (16, 17), which can mobilize splenic immune cells towards to the heart (18).

In this context, the pivotal role of monocytes in cardiac remodeling has been more and more recognized (19, 20). Ly6C monocytes can be divided into two main subsets: pro-inflammatory Ly6C^high^ and anti-inflammatory Ly6C^low^ monocytes. Their recruitment is mediated by different chemokines, whereby CCL2 and CCL7 mediate the mobilization of Ly6C^high^ monocytes (21), and Cx3CL1 governs Ly6C^low^ monocyte attraction (22). Own data support the relevance of these chemokines, since ablation of CX3CR1 resulted in an increased level of CCL2 accompanied by higher numbers of monocytes/macrophages in the heart of viral-infected mice (23). Accumulating evidence shows that cFB influence the migration of splenic pro- (Ly6C^high^) and anti- (Ly6C^low^) inflammatory monocytes, depending on the inflammatory milieu (17, 24). However, the impact of sex and age in this context has not been investigated so far. Furthermore, most studies are directed at evaluating sex- or age-depending effects, whereas the evaluation of age-dependent sex influences are scarce.

Therefore, the aim of the present study was to evaluate the influence of sex and age on the inflammatory potential of cFB and their impact on the cardiosplenic axis and cardiac remodeling.

## 2. Materials and methods

### 2.1. Animals

To evaluate the impact of sex and age on myocardial remodeling and inflammation, 3- and 12-months old male and female C57BL/6JRj mice were obtained from Janvier (Saint Berthevin, France). Animals were housed under standard housing conditions with free excess to water and food *ad libitum*. At the respective time points, mice were sacrificed via cervical dislocation. Afterwards, blood and LV were collected. The LV was immediately snap frozen in liquid nitrogen and stored at −80°C until further examinations. Blood was further processed to obtain plasma used for ELISAs. Additionally, LV-derived cFB were obtained by outgrowth culture from tissue biopsies stored on ice, followed by their basic characterization at passage 0. For flow cytometry, the spleen was harvested and stored on ice until cell isolation. Due to the limited amount of available tissue material, the respective n-number of each group can vary between the different molecular investigations. Therefore, the final n-number is indicated in the respective figure legend. All procedures were in accordance with the European Directive 2010/63/EU for animal welfare and approved by the local ethics committee (Landesamt für Gesundheit und Soziales, Berlin, T0098/11 und T0025/15).

### 2.2. Isolation and expansion of cardiac fibroblasts

Murine cFB were obtained via outgrowth culture as previously described (17, 24). In brief, the LV was cut in small pieces and fixed in 12-well culture plates. Outgrowing cells were cultured in Dulbecco's modified eagle high glucose medium (DMEM) with phenol red (Gibco; Life Technologies, Darmstadt, Germany) containing 20% fetal bovine serum (FBS; Bio&Sell, Feucht, Germany), 1% penicillin (P)/streptomycin (S) (Gibco; Life Technologies) for 4 weeks. Subsequently, cFB were harvested for gene expression analysis at passage 0 or further cell culture maintenance. In addition, supernatant of passage 0 was collected for ELISAs and migration assays. After passage 0, cells were cultured in DMEM media without phenol red (Gibco; Life Technologies) supplemented with 20% FBS (Bio&Sell) and 1% P/S (Gibco; Life Technologies). Subsequent experiments to study cellular senescence or collagen production were performed at passages between 1 and 4.

### 2.3. Evaluation of cellular senescence

To test whether there are differences in cellular senescence between cFB derived from male vs. female mice, a β-galactosidase assay (Cell Signaling Technology Europe B.V., Frankfurt am Main; Germany) was performed. To this end, 7,500 cells/well were seeded in a 96-well-flat-bottom plate at passage 3. At the next day, cFB were washed once with 1xPBS (life technologies). Afterwards, DMEM media without phenol red (Gibco; life technologies) supplemented with 0.5% FBS (Bio&Sell) and 1% P/S (Gibco; life technologies) was added for 24 h. After reaching the end of the stimulation time, cells were again washed once with 1xPBS (life technologies) followed by β-galactosidase staining. Staining procedure was performed according to the manufactures protocol. To image the β-galactosidase staining, a BZ-X800E microscope (Keyence; Neu-Isenburg; Germany) was used. Pictures of *n* = 6 wells/group were taken at 1x or 4x magnification.

### 2.4. RNA isolation, cDNA synthesis and real-time PCR of cardiac fibroblasts

At passage 0, outgrowing cFB were lysed in RLT buffer containing 1% β-mercaptoethanol (Qiagen, Hilden, Germany). Further processing of the samples was performed using the RNeasy® Mini Kit (Qiagen) according to the manufactures protocol (17). Next, 250 ng of total RNA was reverse transcribed using the high-capacity kit (Applied Biosystems, Darmstadt, Germany). To assess mRNA expression of the target genes, real-time PCR was performed on a ViiA7 or QuantStudio6 flex system (both Applied Biosystems) using commercially available gene expression assays (all Applied Biosystems). Following gene expression assays were used: CCL2 (Mm99999056_m1), CCL7 (Mm00443113_m1), Cx3CL1 (Mm00436454_m1), ICAM-1 (Mm00516023_m1), TNF-*α* (Mm00443258_m1), and VCAM-1 (Mm01320970_m1). For relative quantification, detected Ct-values were normalized to CDKN1b (Mm00438167_g1) as internal control by using the 2^−*Δ*Ct^ formula.

### 2.5. ELISA

To determine protein expression of pro- and anti-inflammatory chemokines on cellular or systemic level, ELISAs of cell culture supernatants derived from outgrowth cFBs as well as of plasma samples were performed. As previously described in detail (17), commercially available kits for CCL2 (Peprotech, Hamburg, Germany), CCL7 (Peprotech), and Cx3CL1 (R&D systems Inc., McKinley Place NE, MN, USA) were used. To this end, microplates were coated overnight with the respective capture antibody (1:400 for CCL2, 1:100 for CCL7, and 1:180 for Cx3CL1). Preparation of the standard series and washing steps were carried out according to the manufacturer's protocol. Incubation with the detection antibody (1:2,000 for CCL2 and CCL7, 1:360 for Cx3CL1) was performed for 2 h. After additional washing steps, the avidin-HRP conjugate (1:2,000 for CCL2 and CCL7, 1:200 for Cx3CL1) was added to each well. For color development, ABTS liquid substrate (Sigma-Aldrich, Munich, Germany) was used for the CCL2 and CCL7 ELISA. In case of the Cx3CL1 ELISA, equal volumes of color reagent A and color reagent B were mixed. Absorbance was measured at 405 nm (CCL2 and CCL7) or 450 nm (CxCL13).

### 2.6. Cell migration assay

To determine the potential of the different cFB-derived supernatants to attract different monocyte populations (Ly6C^high^ vs. Ly6C^low^ monocytes), a cell migration assay (Cell Biolabs, San Diego, USA) was performed. For this purpose, splenocytes of the respective male and female mice, which were also used for cFB generation and supernatant production, were isolated according to Van Linthout et al. (25). In accordance to the manufacturer's protocol, splenocytes were placed at a density of 0.1 × 10^6^ cells/mL on the upper membrane chamber. Next, 150 µl of the respective supernatants were added to the wells of the feeder tray and incubated for 24 h. At the end of the incubation time, migratory cells were harvested and further analyzed via flow cytometry. In total, 2–3 mice/group were used for supernatant harvest and splenocyte isolation. For each mice, *n* = 8 wells were pooled for flow cytometry.

### 2.7. Flow cytometry

Flow cytometry was performed to investigate the different monocyte subsets of the migrated splenocytes in the cell migration assay and to analyze the different monocyte subsets and TGF-ß expressing mononuclear cells in the spleen of male and female mice. Therefore, splenocytes were isolated according to Van Linthout et al. (25). To determine pro-inflammatory (CD11b^+^ CD115^+^ Ly6C^high^) and patrolling/reparative (CD11b^+^ CD115^+^ Ly6C^low^) monocytes, migrated or isolated splenocytes were directly stained without polyclonal stimulation. For this purpose, anti-CD11b, anti-CD115, and anti-Ly6C antibodies were purchased from Biolegend (London, UK) (26). For the analysis of TGF-ß-expressing splenic mononuclear cells, isolated splenocytes were plated in Iscove's modified Dulbecco's Medium (Sigma Aldrich, Munich, Germany) supplemented with 10% FBS (Biochrom) and 1% P/S (Biochrom). For polyclonal stimulation, 50 ng/ml phorbol myristate acetate (PMA) and 500 ng/ml ionomycin, in the presence of BD GolgiStopTM protein transport inhibitor (Invitrogen, Thermo Fisher Scientific, Waltham, MA, USA), were added for 12 h. After permeabilization and fixation, intracellular staining was performed with an anti-TGF-β antibody (Biolegend, San Diego, USA) according to (27). After the respective staining, samples were measured on the MACSQuant Analyzer (Miltenyi Biotec) and analyzed via the FlowJo 8.7. Software (FlowJo, LLC, RO, USA).

### 2.8. Co-culture of C4 fibroblasts with splenocytes

To determine the pro-fibrotic potential of the isolated splenocytes, co-culture experiments with murine C4 fibroblasts were performed, as described previously (27–29). In detail, C4 fibroblasts were plated at a density of 10,000 cells/well in Iscove Basal Medium (Sigma-Aldrich) containing 10% FBS and 1% P/S (both Biochrom). One day later, isolated splenocytes were added at a ratio of 1 to 10 (fibroblasts to splenocytes) in cell culture media and removed after 24 h of co-culturing. Subsequently, fibroblasts were fixed with cold methanol (Carl Roth, Karlsruhe, Germany) and Sirius red staining (Sigma-Aldrich Chemie GmbH) was performed. Collagen production in the C4 fibroblasts was photometric analyzed via the Spectra Max 340PC microplate reader (Molecular Device GmbH) at 540 nm (29).

### 2.9. Immunofluorescence staining of left ventricular tissue slides

Acetone-fixed and -permeabilized LV transverse OCT sections, blocked with 1% bovine serum albumin in PBS, were stained with mouse anti-α-actinin (A7811, Sigma-Aldrich; secondary antibody: FITC donkey anti-mouse, 715-096-150, JacksonImmunoResearch, West Grove, PA, USA), rat anti-mouse CD68 (MCA 1957GA, Bio-Rad; Hercules, CA, USA; secondary antibody: Alexa Fluor 647 Donkey anti-rat IgG, 712-605-153, JacksonImmunoResearch) and goat anti-mouse CD206 (AF2535, R&D, Wiesbaden, Germany; secondary antibody: Rhodamine Red-X Donkey anti-goat, 705-295-147, JacksonImmunoResearch). Nuclei were stained with Hoechst (H3570, Invitrogen, Waltham, MA, USA). Macrophage populations were manually counted (Image J) in 2 non-consecutive sections per heart in 4–5 fields of view (FoV) per section, acquired in 20x magnification, and averaged per mouse. CD68^+^ CD206^+^ and CD68^+^ CD206^−^ subpopulations were quantified as percentage (%) of CD68^+^ cells. A schematic illustration of the acquisition and quantification procedure is depicted in the supplement (Supplementary Figure S1). For representative pictures, which are shown in Figure 5D, the 40x magnification was used.

### 2.10. Immunohistological staining

For immunohistological stainings, frozen LV tissue was embedded in Tissue-Tek (Sakura, Staufen, Germany) and cut into 5 µm-thick cryosections. Subsequently, slides were stained with antibodies directed against Collagen I (Chemicon, Limburg, Germany) and Collagen III (Calbiochem, Merck Millipore, Darmstadt, Germany) (30). Analysis and quantification were performed at a 200x magnification using the LAS Software (Leica Microsystems, Wetzlar, Germany). Data were expressed as positive area (%) per heart area (HA; in mm^2^).

### 2.11. Determination of the collagen content of cardiac fibroblasts

To determine the collagen content of cFB derived from male and female mice at different ages, a Sirius Red and crystal violet assay was performed at passage 1. As previously described (31, 32), cFB of 3 mice/group with *n* = 3–8 wells/mice were seeded at a cell density of 7,500 cells/well in two 96-well plates. After 72 h, media was removed and one plate was fixated with cold methanol (Carl Roth) for Sirius red staining (Sigma-Aldrich Chemie GmbH) to measure collagen amount in cFB. The second plate was fixated with 4% paraformaldehyde (PFA; SAV Liquid Production GmbH, Flintsbach am Inn, Germany) for crystal violet staining (Sigma-Aldrich Chemie GmbH) to determine the cell number/well. For photometric analyses, the Spectra Max 340PC microplate reader (Molecular Device GmbH LLC, San Jose, CA, USA) was used and absorbance was measured at 540 nm or 595 nm. Absorbance values were depicted separately (collagen amount and cell count) or normalized (collagen amount/cell).

### 2.12. Statistical analysis

Data are depicted as scatter plots with bars, showing individual data points and the respective mean ± SEM. For statistical analyses, mean ± SEM were analyzed with Two-way-ANOVA (full model to determine column effect, row effect, and column/row interaction effect) with Fisheŕs LSD *post hoc* test. Differences between the groups were assessed significant at a *p*-value lower than 0.05. All Statistical analyses were performed via the GraphPad Prism 9.5.1 software (GraphPad Software Inc, La Jolla, USA).

## 3. Results

### 3.1. Sex- and age-related changes in the inflammatory capacity of cardiac fibroblasts

**Figure 1:**
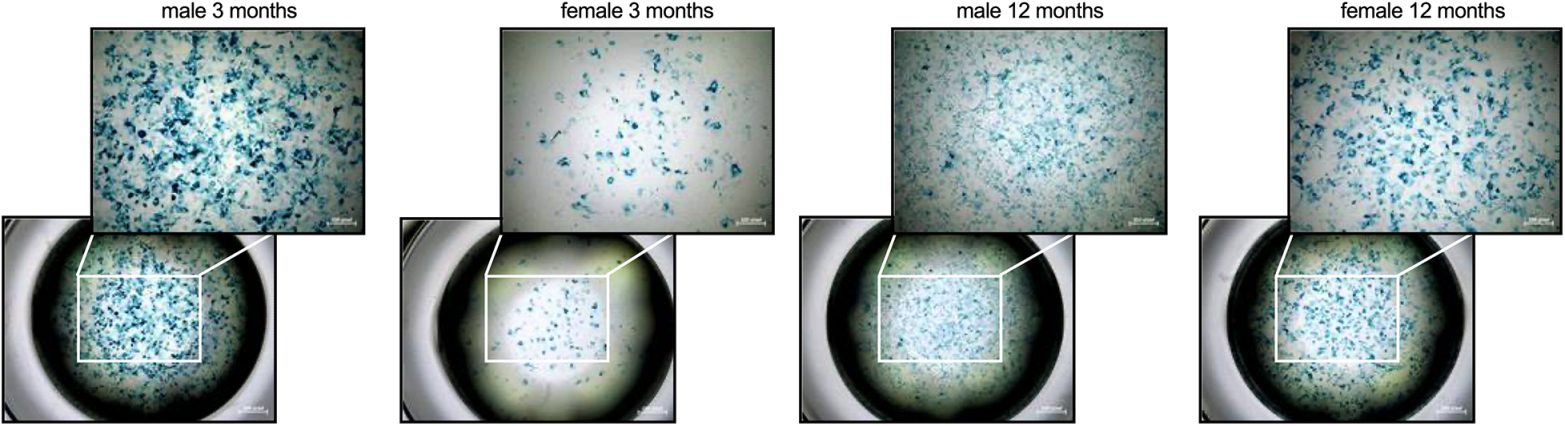
Sex- and age-related differences in cellular senescence of cardiac fibroblasts. To determine if there are differences in cellular senescence, a β-galactosidase assay at passage 3 was performed. In detail, senescent cells are characterized by the expression of pH-dependent β-galactosidase activity as shown by the accumulation of the blue dye. The figure shows representative images of each group at1x (lower panel) or 4x magnification (upper panel; scale bar = 200 pixel).

**Figure 2:**
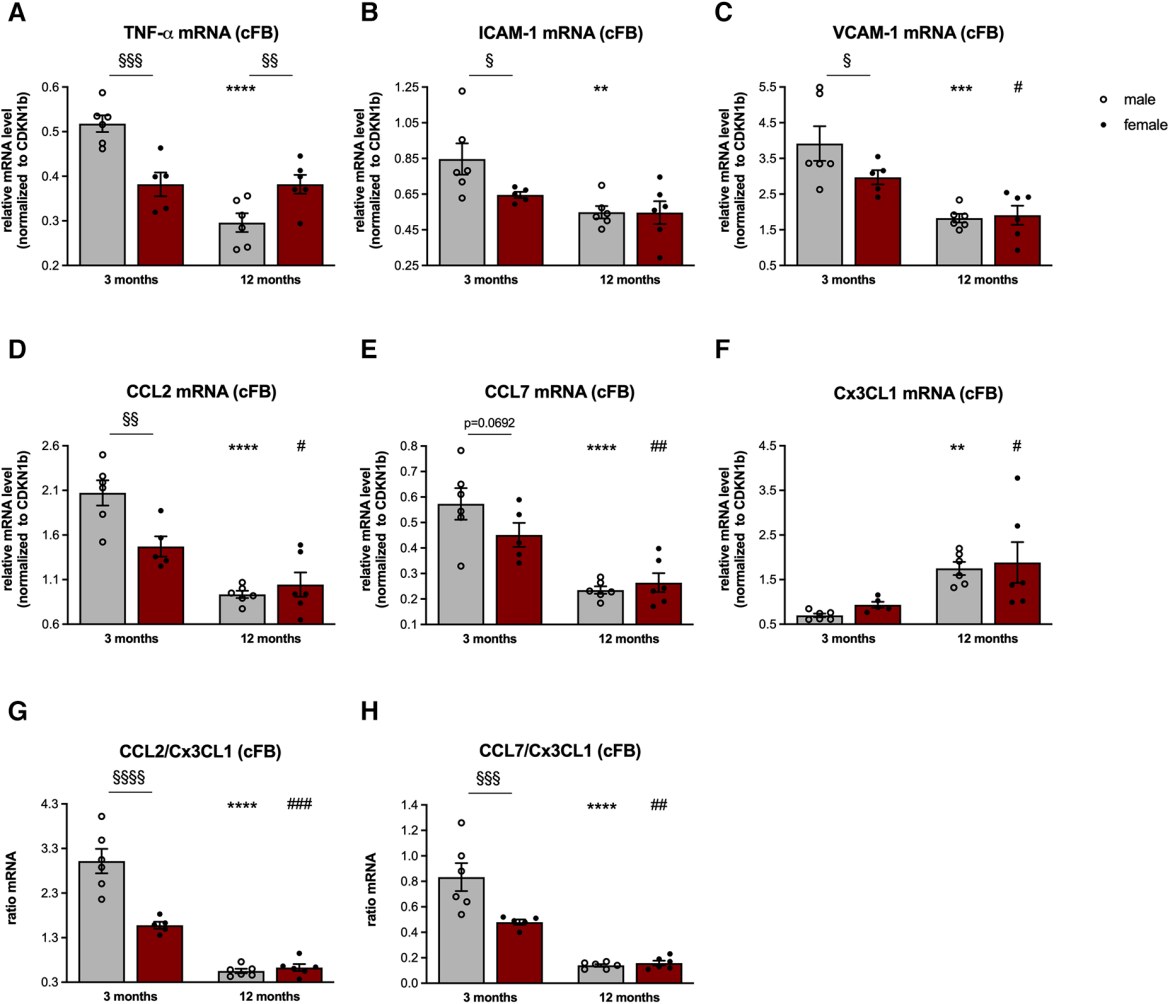
Sex- and age-related changes in the inflammatory capacity of cardiac fibroblasts. To determine the inflammatory capacity of male and female LV-derived fibroblasts at the age of 3- and 12-months, gene expression of the pro-inflammatory genes tumor necrosis factor (TNF)-*α* (**A**), intercellular adhesion molecule (ICAM)-1 (**B**), vascular cell adhesion molecule (VCAM)-1 (**C**), CC-chemokine ligand (CCL) 2 (**D**), CCL7 (**E**) and chemokine C-X3-C motif ligand (Cx3CL1; **F**) was analyzed. Next, the CCL2/Cx3CL1 (**G**) and CCL7/Cx3CL1 (**H**) ratiós were calculated as parameters to estimate the attraction of splenic monocyte subsets towards the heart. Data are depicted as scatter plots with bars (male mice: gray bars; female mice: wine red bars), showing individual data points and the corresponding mean ± SEM. For statistical analysis, Two-way ANOVA with Fisheŕs LSD *post hoc* test was performed (^§^*p* < 0.05, ^§§^*p* < 0.01, ^§§§^*p* < 0.001, ^§§§§^*p* < 0.0001 male vs. female mice; *p < 0.05, **p < 0.01, ***p < 0.001, ****p < 0.0001 vs. 3-months old male mice; ^#^*p* < 0.05, ^##^*p* < 0.01, ^###^*p* < 0.001, ^####^*p* < 0.0001 vs. 3-months old female mice; with *n* = 6 for 3-months old male mice, *n* = 5 for 3-months old female mice, *n* = 6 for 12-months old male mice, *n* = 6 for 12-months old female mice).

Since cFB are recognized as important inflammatory supporter cells (13, 16, 17, 24) and senescent cells are able to switch into a senescence-associated secretory phenotype (SASP) with inflammatory potential (33, 34), cellular senescence (Figure 1) and the inflammatory capacity of male and female cFB was analyzed (Figure 2).

In detail, cFB derived from 3-months old male mice were characterized by a higher β-galactosidase activity compared to age-matched female cFB, indicative for senescence (Figure 1). In accordance with an inflammatory phenotype, 3-months old male cFB displayed a 1.4-fold (*p* < 0.001), 1.3-fold (*p* < 0.05) and 1.3-fold (*p* < 0.05) higher gene expression of the pro-inflammatory genes TNF-*α* (Figure 2A), ICAM-1 (Figure 2B), and VCAM-1 (Figure 2C), respectively, compared to age-matched female cFB. Additionally, gene expression of chemokines, which are known to attract different monocyte subsets (22), were measured. Compared to cFB from 3-months old female mice, cFB derived from male mice displayed 1.4-fold (*p* < 0.01) and 1.3-fold (*p* = 0.0692) higher mRNA levels of CCL2 (Figure 2D) and CCL7 (Figure 2E), whereas no difference in gene expression of Cx3CL1 (Figure 2F) was observed. This resulted in a 1.9-fold (*p* < 0.0001) and 1.7-fold (*p* < 0.001) higher CCL2/Cx3CL1 (Figure 2G) and CCL7/Cx3CL1 (Figure 2H) ratio in 3-months old male vs. female cFB. In contrast to the prominent sex differences in gene expression of pro-inflammatory genes and chemokines observed in cFB derived from 3-months old mice, only a sex-dependent difference in TNF-*α* mRNA was found in cFB of 12-months old mice (Figure 2A).

### 3.2. Sex- and age-related modulation of splenic monocyte migration

**Figure 3:**
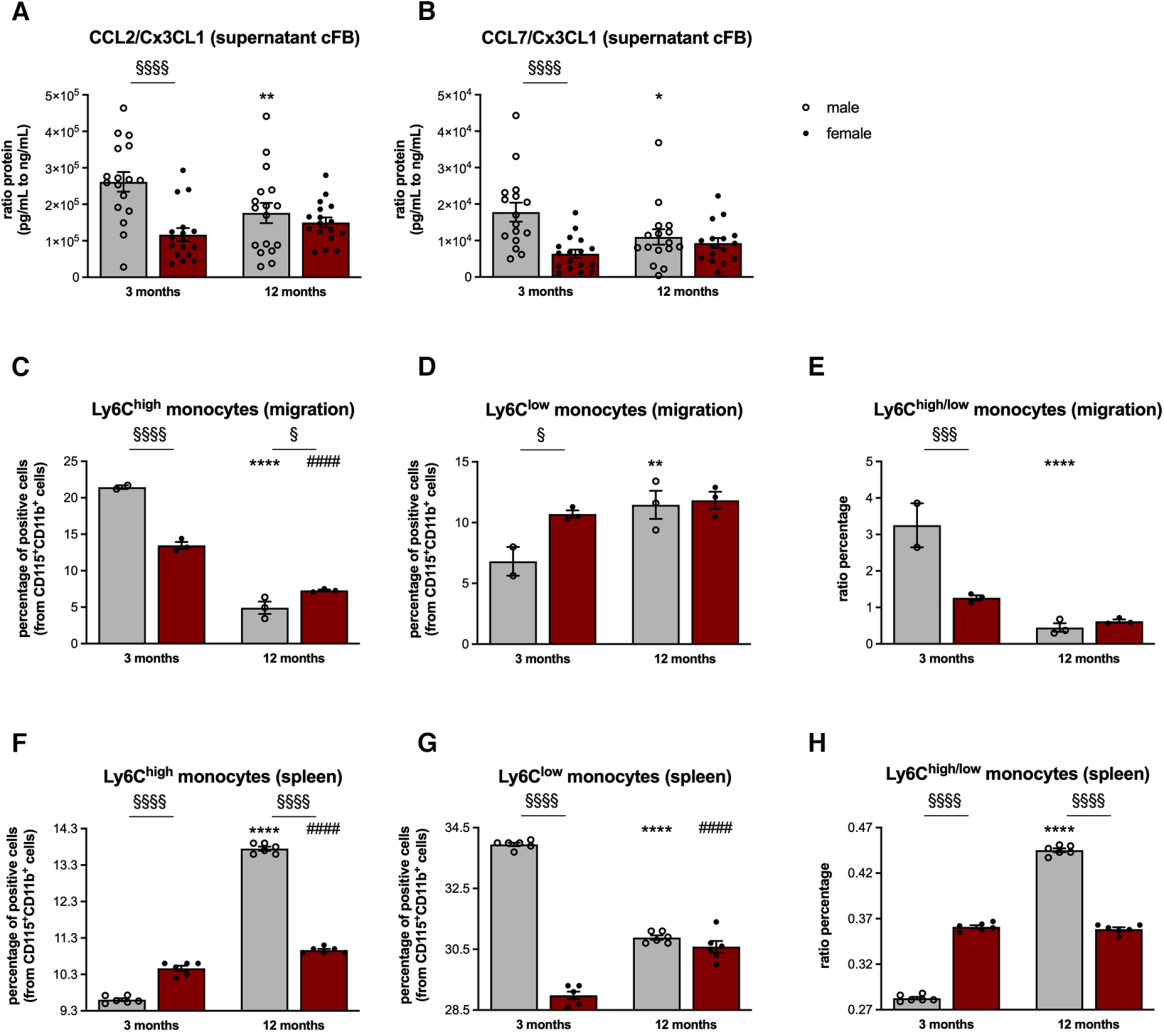
Sex- and age-dependent modulation of monocyte migration. In order to investigate the chemotactic potential of cFB derived from male and female mice, analysis of the CCL2, CCL7, and Cx3CL1 protein concentration in cFB-derived supernatant was performed (with *n* = 5-6/group and *N* = 3). Subsequently, CCL2/Cx3CL1 (**A**) and CCL7/Cx3CL1 (**B**) ratios were calculated as parameters to estimate the attraction of splenic monocyte subsets towards the heart. To further substantiate this, cell migration assays of splenocytes towards the respective supernatant of the corresponding cFB were performed. The fraction of migrated pro-inflammatory (CD11b^+^ CD115 + Ly6C^high^; **C**) and patrolling/reparative (CD11b^+^ CD115^+^ Ly6C^low^; **D**) monocytes and the ratio thereof (**E**) was determined via flow cytometry (with 2-3 mice/group for supernatant harvest and splenocyte isolation; *n* = 8 wells/mice were pooled for flow cytometry analysis). Finally, the percentage of monocytes in the spleen of 3- and 12-months old mice was analyzed via flow cytometry (**F,G**; with *n* = 6/group). In addition, the ratio of splenic Ly6C^high^ to Ly6C^low^ monocytes was calculated (**H**) Data are depicted as scatter plots with bars (male mice: gray bars; female mice: wine red bars), showing individual data points and the corresponding mean ± SEM. For statistical analysis, Two-way ANOVA with Fisheŕs LSD *post hoc* test was performed (^§^*p* < 0.05, ^§§^*p* < 0.01, ^§§§^*p* < 0.001, ^§§§§^*p* < 0.0001 male vs. female mice; *p < 0.05, **p < 0.01, ***p < 0.001, ****p < 0.0001 vs. 3-months old male mice; ^#^*p* < 0.05, ^##^*p* < 0.01, ^###^*p* < 0.001, ^####^*p* < 0.0001 vs. 3-months old female mice).

Given the relevance of the cardiosplenic axis in cardiac fibrosis on the one hand (20) and the potential of cFB to attract different monocyte subsets depending on the inflammatory milieu on the other hand (17, 24), we next investigated the CCL2, CCL7, and Cx3CL1 protein concentration in cFB-derived supernatant (Figures 3A,B).

In line with the gene expression data, cFB derived from 3-months old male mice displayed a 2.2-fold (*p* < 0.0001) and 2.8-fold (*p* < 0.001) higher CCL2/Cx3CL1 and CCL7/Cx3CL1 protein ratio compared to the age-matched female cFB. Additional normalization of the protein concentration against the protein content of the cFB lysates resulted in a 1.5-fold (*p* < 0.01) and 1.6-fold (*p* < 0.05) higher CCL2/Cx3CL1 and CCL7/Cx3CL1 protein ratio in the supernatant of male cFB vs. female cFB of 3-months old animals (Supplementary Figure S2).

This higher pro-inflammatory potential of male cFB at the age of 3-months was further reflected by a higher percentage of migrated pro-inflammatory Ly6C^high^ monocytes towards supernatant from cFB derived from 3-months old male mice compared to supernatant derived from age-matched female cFB (Figure 3C). In addition, the fraction of migrated Ly6C^low^ monocytes was lower towards the supernatant derived from 3-months old male cFB compared to female cFB (Figure 3D), resulting in a higher ratio of Ly6C^high/low^ monocytes for cFB from 3-months old male vs. female mice (Figure 3E).

Although no differences in circulating levels of CCL2, CCL7, and Cx3CL1 were observed between the male and female mice (Supplementary Figure S3), the differences in monocytes migration towards the cell culture supernatants of cFB were also reflected by changes of splenic monocyte subsets *in vivo* (Figures 3F,H). In detail, 3-months old female mice displayed 1.1-fold (*p* < 0.0001) higher pro-inflammatory Ly6C^high^ monocytes (Figure 3F), and 1.2-fold (*p* < 0.0001) lower anti-inflammatory Ly6C^low^ monocytes compared to age-matched male mice (Figure 3G). This resulted in a higher ratio of splenic Ly6C^high^ to Ly6C^low^ monocytes in 3-months old female vs. male mice (Figure 3H). At the age of 12-months, the number of splenic Ly6C^high^ monocytes was lower in female vs. male mice (Figure 3F), whereas the number of Ly6C^low^ monocytes did not differ between the sexes (Figure 3G), leading to a lower Ly6C^high/low^ monocyte ratio in 12-months old female compared to male mice (Figure 3H). This is indicative for a higher migration of Ly6C^high^ monocytes towards the heart in female mice during aging, which is also reflected by a higher number of migrated Ly6C^high^ monocytes towards the supernatant of female cFB at the age of 12-months (Figure 3C).

### 3.3. Sex- and age-dependent pro-fibrotic potential of isolated splenocytes

**Figure 4:**
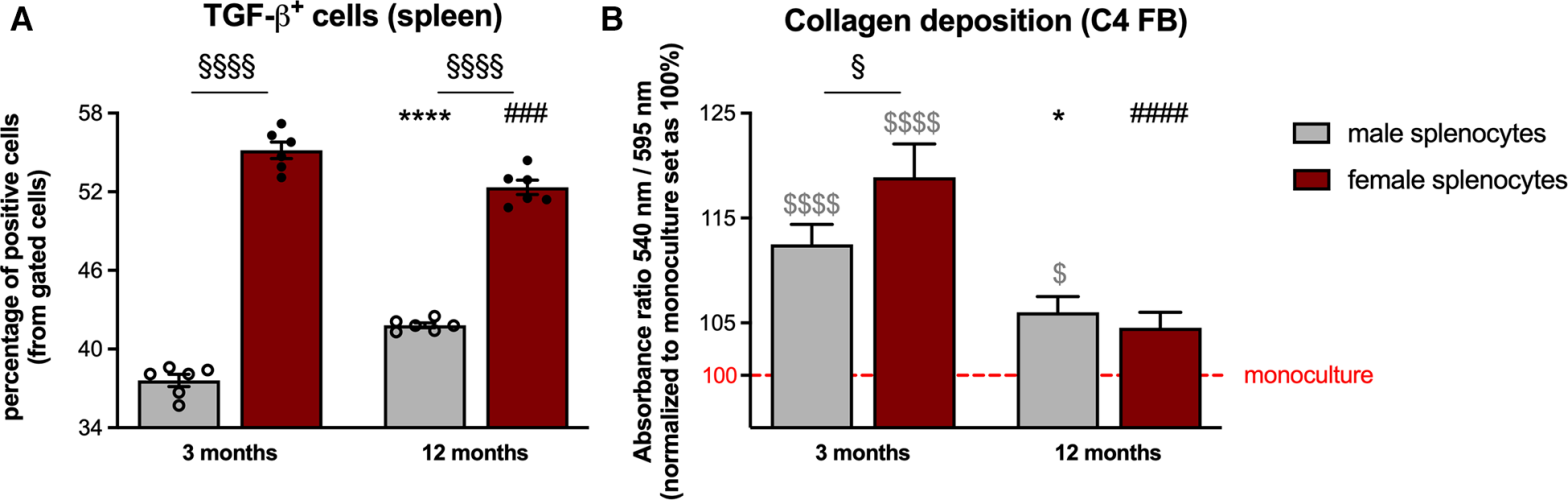
Sex- and age-dependent pro-fibrotic potential of isolated splenocytes. TGF-β expression of splenocytes from male and female mice was analyzed via flow cytometry [(**A**) with *n* = 6/group]. In parallel, the pro-fibrotic potential of the splenocytes was investigated via different co-culture experiments. In detail, collagen production in C4 fibroblasts was measured via Sirius red staining after 24 h of co-culture with the respective splenocytes [(**B**) with *n* = 34–36/group]. Data are depicted as scatter plots with bars (male mice: gray bars; female mice: wine red bars), showing individual data points and the corresponding mean ± SEM. For statistical analysis, Two-way ANOVA with Fisheŕs LSD *post hoc* test was performed (^§^*p* < 0.05, ^§§^*p* < 0.01, ^§§§^*p* < 0.001, ^§§§§^*p* < 0.0001 male vs. female mice; *p < 0.05, **p < 0.01, ***p < 0.001, ****p < 0.0001 vs. 3-months old male mice; ^#^*p* < 0.05, ^##^*p* < 0.01, ^###^*p* < 0.001, ^####^*p* < 0.0001 vs. 3-months old female mice). To analyze the differences between the collagen production under monoculture conditions, ordinary One-way ANOVA with Fisheŕs LSD *post hoc* test was performed (^$^*p* < 0.05, ^$$$$^*p* < 0.0001 vs. monoculture).

With the importance of splenocytes in cardiac remodeling (20, 22) on the one hand, and the above reported modulation of the splenic monocyte composition on the other hand, we next examined the pro-fibrotic potential of splenocytes derived from the different groups (Figure 3). Analysis of splenic TGF-*β*^+^ cells revealed a higher percentage thereof in both 3- and 12-months old female mice compared to male animals (Figure 4A). This was further reflected by a higher pro-fibrotic potential of female vs. male splenocytes at both ages. In detail, collagen production was increased in C4 fibroblasts supplemented with splenocytes derived from 3-months old male and female mice (Figure 4B), whereby this increase was more pronounced upon co-culture with female splenocytes.

### 3.4. Sex- and age-dependent presence of cardiac macrophages

**Figure 5:**
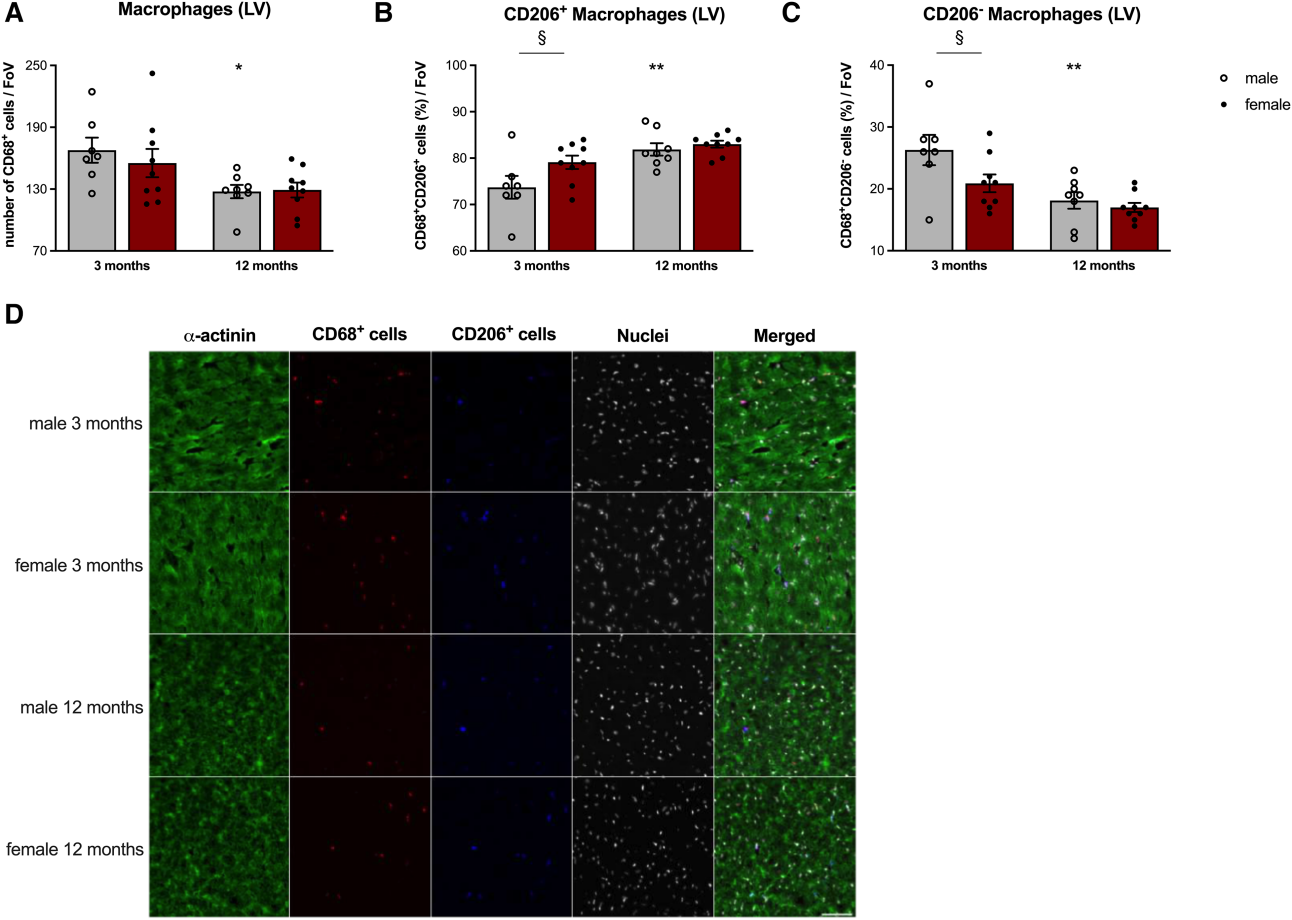
Sex- and age-dependent differences in left ventricular macrophage presence. To determine LV presence of CD68^+^ macrophages (**A**), including the anti-inflammatory CD68^+^ CD206^+^ (**B**) and pro-inflammatory CD68^+^ CD206^−^ (**C**) subsets, immunofluorescence on tissue sections was performed. After the respective stainings, pictures were acquired at 20x magnification and macrophage populations were manually counted per Field of View (FoV). Amount of CD68^+^ cells were expressed as number of cells/FoV. CD68^+^ CD206^+^ and CD68^+^ CD206^−^ subpopulations were quantified as percentage (%) of CD68^+^ cells. Representative images [(**D**); 40x magnification; scale bar = 50 *μ*m] illustrate the different stainings on LV cryosections of male and female mice at the age of 3- and 12-months. Data are depicted as scatter plots with bars (male mice: gray bars; female mice: wine red bars), showing individual data points and the corresponding mean ± SEM. For statistical analysis, Two-way ANOVA with Fisheŕs LSD *post hoc* test was performed (^§^*p* < 0.05, ^§§^*p* < 0.01, ^§§§^*p* < 0.001, ^§§§§^*p* < 0.0001 male vs. female mice; *p < 0.05, **p < 0.01, ***p < 0.001, ****p < 0.0001 vs. 3-months old male mice; with *n* = 7 for 3-months old male mice, *n* = 9 for 3-months old female mice, *n* = 8 for 12-months old male mice, *n* = 9 for 12-months old female mice).

Given the above reported lower CCL2/Cx3CL1 and CCL7/Cx3CL1 ratio in 3-months old female vs. male cFB, accompanied by the lower Ly6C^high^ monocyte migration and higher splenic Ly6C^high/low^ monocyte ratio, we next determined the presence of pro- and anti-inflammatory macrophages in the LV of 3- and 12-months old animals (Figures 5A–D). Immunofluorescence staining revealed a lower number of CD68^+^ monocytes/macrophages (Figure 5A) in old male mice compared to young males. Determination of the anti-inflammatory CD68^+^ CD206^+^ macrophages (Figure 5B) indicated a higher number of this subset in 3-months old female mice compared to age-matched male animals. Along with this, the percentage of the pro-inflammatory counterparts (CD68^+^ CD206^−^ cells; Figure 5C) was lower in female vs. male mice at the age of 3-months. Interestingly, changes in the percentage of both subsets due to aging were only detected in the LV of male mice.

### 3.5. Sex- and age-related alterations in left ventricular fibrosis

**Figure 6:**
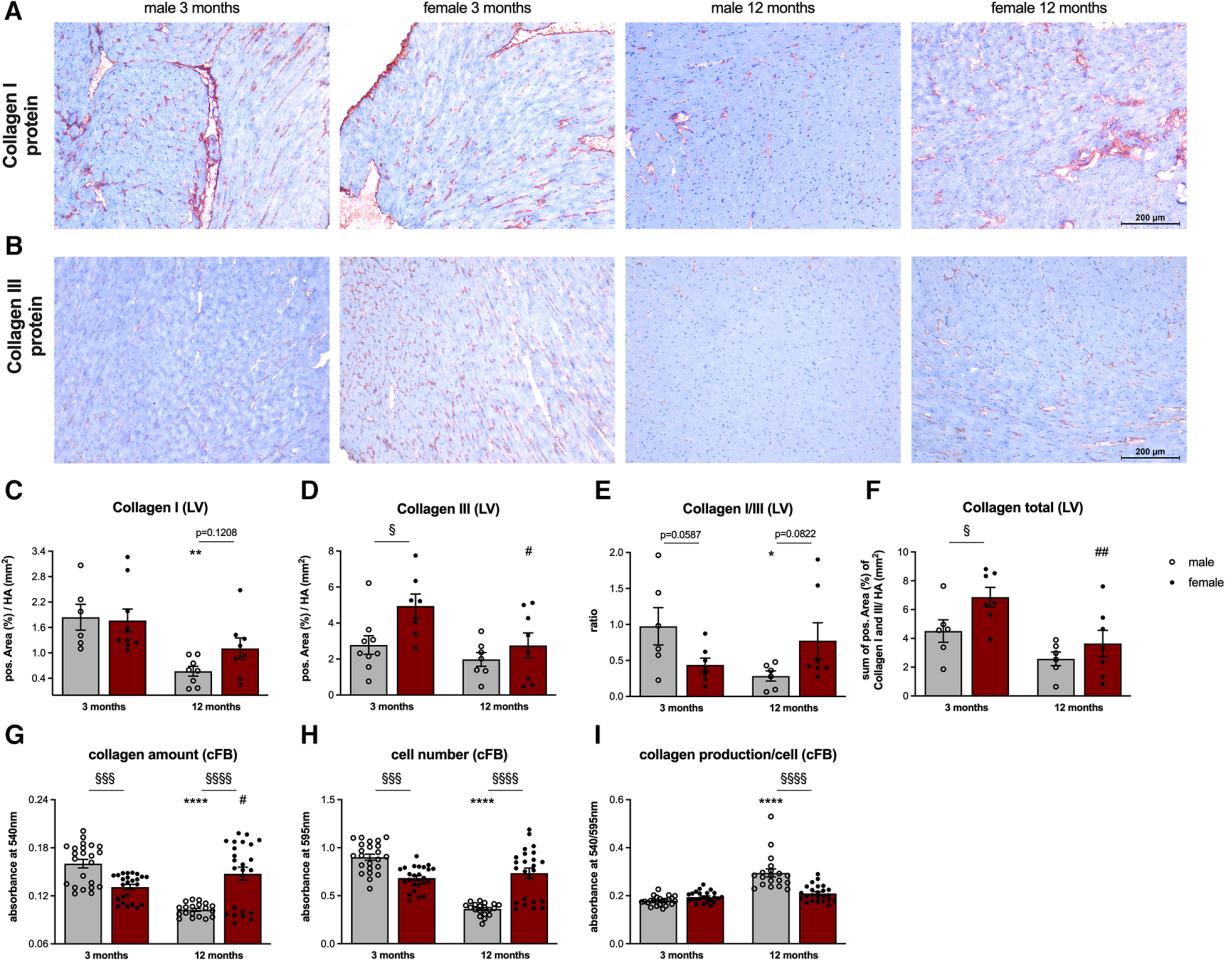
Sex- and age-related alterations in left ventricular fibrosis and collagen content of cardiac fibroblasts. Representative images (scale bar = 100 *μ*m) of left ventricular (LV) collagen I (**A**) and collagen III (**B**) staining and the corresponding quantification of the positive area (%)/heart area (HA, mm^2^; **C, D**). Additionally, the collagen I/III ratio (**E**) and total collagen content (**F**) were calculated. To analyze the collagen content of cFB derived from male and female mice at different ages, a Sirius Red (SR) and crystal violet (CV) assay at passage 1 were performed. SR staining was used to measure collagen amount/well (**G**) and the CV staining to determine the cell number/well (**H**) Subsequently, ratio of the respective absorbance values was calculated to determine the collagen production/cell (**I**). Data are depicted as scatter plots with bars, showing individual data points and the corresponding mean ± SEM. For statistical analysis, Two-way ANOVA with Fisheŕs LSD *post hoc* test was performed (^§^*p* < 0.05, ^§§^*p* < 0.01, ^§§§^*p* < 0.001, ^§§§§^*p* < 0.0001 male vs. female mice; *p < 0.05, **p < 0.01, ***p < 0.001, ****p < 0.0001 vs. 3-months old male mice; ^#^*p* < 0.05, ^##^*p* < 0.01, ^###^*p* < 0.001, ^####^*p* < 0.0001 vs. 3-months old female mice; with *n* = 6–7 for 3-months old male mice, *n* = 7–9 for 3-months old female mice, *n* = 6–9 for 12-months old male mice, *n* = 7–9 for 12-months old female mice for IHC staining and *N* = 3 with *n* = 3–8 wells/mice for SR and CV assay).

Immunohistochemistry of collagen I (Figure 6A) and III (Figure 6B) revealed no differences in LV collagen I protein expression between male and female 3-months old mice (Figure 6C). In contrast, LV collagen III protein level was 1.8-fold (*p* < 0.05) higher in female vs. male animals (Figure 6D) leading to a trend toward a lower collagen I/III protein ratio in female animals at this age (Figure 6E). With respect to the total collagen amount, 3-months old female animals displayed a 1.5-fold (*p* < 0.05) higher collagen content compared to male mice (Figure 5F). Interestingly, collagen I expression in 12-months old female mice was 2.0-fold (*p* < 0.01) higher compared to age-matched male animals (Figure 6C). Since collagen III protein expression did not differ between the sexes (Figure 6D), the collagen I/III protein ratio tended to be higher in 12-months old female vs. male mice (Figure 6E). Despite not reaching statistical significance, total collagen content also tended to be higher in female vs. male mice at this age (Figure 6F).

Finally, differences in collagen deposition between male and female mice were examined on cellular level (Figures 6G–I). Under basal conditions, collagen amount in cFB derived from 3-months old male mice was 1.2-fold (*p* < 0.001) higher compared to cFB derived from 3-months old female mice (Figure 6G). Taking the cell number into account, collagen production per cell differed between male and female cFB derived from 3-months old animals (Figure 6I). Interestingly, female cFB derived from 12-months old animals displayed a 1.4-fold (*p* < 0.0001) and 2.0-fold (*p* < 0.0001) higher collagen amount and cell number compared to the age-matched male cFB, respectively (Figure 6G,H). Subsequent normalization revealed 1.4-fold (*p* < 0.0001) lower collagen production per cell in old female cFB compared to old male cFB.

## 4. Discussion

**Figure 7:**
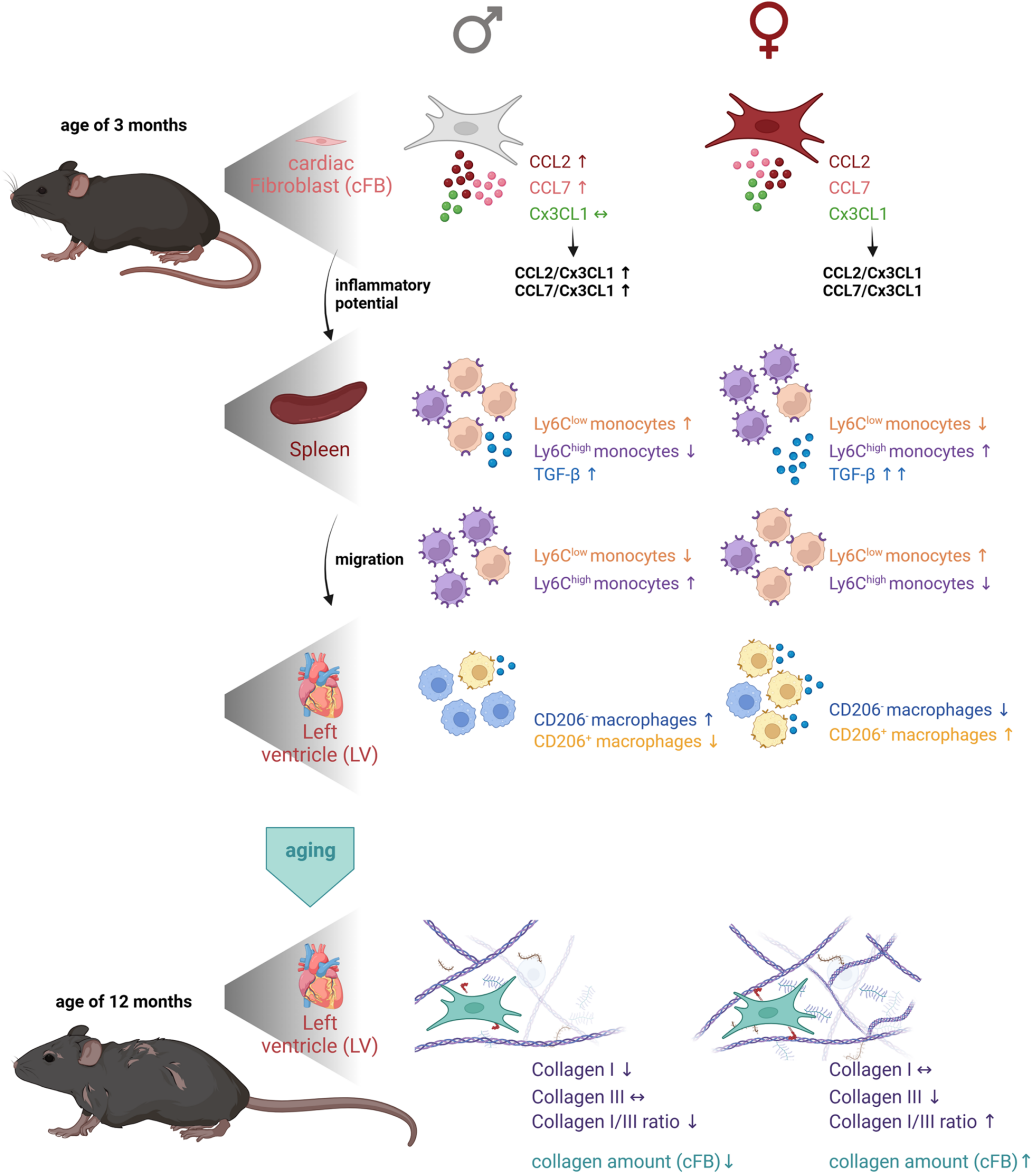
Graphical abstract representing the impact of cardiac fibroblasts as central players in sex- and age-related differences in cardiac fibrosis and inflammation. The first panel of the figure illustrates the higher inflammatory potential of male cFB vs. female cFB at the age of 3-months. Analysis of the spleen revealed a higher number of anti-inflammatory Ly6C^low^ monocytes and a lower number of pro-inflammatory Ly6C^high^ monocytes in the spleen of male vs. female animals, supporting the hypothesis that due to the higher inflammatory potential of male cFB, the pro-inflammatory Ly6C^high^ monocytes migrated towards the heart. In contrast, cFB derived from female mice have a lower inflammatory potential mediating migration of pro-inflammatory Ly6C^high^ monocytes towards the heart, as reflected by a higher number of pro-inflammatory Ly6C^high^ monocytes in the spleen of female vs. male animals. In agreement, the percentage of pro-inflammatory CD206^−^ macrophages was higher in the LV of males compared to females, whereas the number of anti-inflammatory (pro-fibrotic) CD206^+^ macrophages was higher in females vs. male mice. By aging, the collagen I/III ratio in the LV was higher in female mice compared to male mice, which was associated with a higher collagen content in 12-months old female compared to male cFB. The Figure was created with BioRender.com.

The present study reveals age- and sex-dependent differences in the inflammatory competence of cFB, and modulation of the cardiosplenic axis and cardiac fibrosis. In detail, we detected in young animals a higher inflammatory capacity of male cFB compared to female cFB, as indicated by higher levels of the chemokines CCL2 and CCL7. In addition, young male mice displayed a lower ratio of Ly6C^high^ to Ly6C^low^ monocytes in the spleen compared to female animals. Subsequent analysis of cardiac anti- and pro-inflammatory macrophages revealed a higher number of the pro-inflammatory subset in the LV of young male vs. female mice, whereas the number of anti-inflammatory macrophages was higher in females vs. males. During aging, sex-dependent modulation of cardiac fibrosis took place and resulted in a higher collagen I/III ratio in the heart of female mice vs. male animals at the age of 12-months, whereas total collagen deposition was higher in female vs. male mice, independent of age. We hypothesize that the sex-dependent differences in the inflammatory capacity of cFB influence the splenic monocyte retention and the migration of monocytes towards the heart, which further modulates cardiac ECM production (Figure 7).

In general, it is accepted that with increasing age, cardiac fibrosis and tissue remodeling occur, leading to an increased stiffening of the heart (4, 35, 36). LV fibrosis is caused by excessive deposition of ECM components, whereby cFB are the main producers as well as regulators (13, 37). Beyond ECM production, cFB act as important modulators of inflammation (13, 16, 17, 24). So far, only a few studies have simultaneously examined age- and sex-related differences in cardiac fibrosis (38–43). In order to close this knowledge gap, we studied the influence of aging and sex on cardiac fibrosis and inflammation in healthy mice on cellular and tissue level.

Twelve months old male mice displayed lower protein levels of the stiff collagen I compared to 3-months old male mice, whereas 12-months old female mice showed lower protein levels of the compliant collagen III vs. 3-months old female mice. This resulted in a higher collagen I/III ratio in 3-months old male vs. female animals, and a shift to a higher collagen I/III ratio in 12-months old female vs. male mice, suggesting that 12-months old female mice exhibit stiffer hearts than age-matched male mice. On cellular level, a similar phenomenon could be observed by which young female cFB exhibited lower collagen content compared to age-matched male cFB, but 12-months old female cFB displayed higher collagen vs. 12-months old male cFB. Independent of sex, total collagen content decreased with increasing age. These data are consistent with findings of Wu et al.*,* who reported lower Col1a1 and Col3a1 gene expression levels in old mice (44) and were confirmed by measurements by other investigators, detecting a lower content of insoluble collagen in the heart of aged animals (45).

Related to the inflammatory capacity of cFB, we and also others (44) could demonstrate that 3-months old male cFB display higher gene expression of inflammatory markers, including the chemokines CCL2 and CCL7, compared to their age-matched female cFB. In agreement with the inflammatory potential of senescent cells (33, 34), we further showed that 3-months old male cFB exhibited a more pronounced senescent phenotype compared to 3-months old female cFB. Since cell density is known to affect cellular senescence (46), one cannot rule out that part of the differences in observed cellular senenscence among the different cFBs is due to differences in cell growth kinetics.

The higher CCL2/Cx3CL1 and CCL7/Cx3CL1 ratio in (supernatant from) male vs. female cFB from 3-months old mice and the existence of the so called cardiosplenic axis by which monocytes home from the spleen to the heart (18), suggest a higher attraction of pro-inflammatory monocytes to male compared to female hearts. This hypothesis was further supported by our data showing higher numbers of migrated pro-inflammatory Ly6C^high^ monocytes and lower numbers of migrated anti-inflammatory Ly6C^low^ monocytes towards the supernatant of 3-months old male vs. female cFB. Adressing the question how CCL2, CCL7 and Cx3CL1 secreted by the cFB specifically contributed to the migration of pro- and anti-inflammatory monocytes was beyond the scope of our investigations and requires further migration assays with respective antibodies. Additionally we could demonstrate lower numbers of pro-inflammatory Ly6C^high^ and higher number of anti-inflammatory Ly6C^low^ monocytes in the spleen of 3-months old male mice, indicative for a retention of Ly6C^low^ monocytes in the spleen. We postulate that in young male mice mainly Ly6C^high^ monocytes migrate towards the heart, whereas in young female mice mainly Ly6C^low^ monocytes home to the heart. In agreement, young female mice exhibited a lower percentage of pro-inflammatory CD68^+^ CD206^−^ and a higher percentage of anti-inflammatory CD68^+^ CD206^+^ macrophages in the LV compared to age-matched male animals. At the age of 12-months, no differences in pro- or anti-inflammatory macrophages were observed in the LV of male and female mice. With anti-inflammatory macrophages (47) being characterized by secretion of anti-inflammatory but also pro-fibrotic mediators, such as TGF-β (48, 49), the higher collagen deposition observed in 3-months and 12-months old female vs. male mice might be in part a reflection of the number of anti-inflammatory macrophages in 3-months old female mice driving the fibrosis process. In this regard, it is further important to point out that female mice exhibited more TGF-ß-expressing splenocytes with higher pro-fibrotic potential than male mice at both ages. This supports the hypothesis that the impact of the cardiosplenic axis on cardiac remodeling is a dynamic process, by which alterations in TGF-ß expressing splenic cells may predict fibrotic processes in the heart (29).

Our findings are based on 3- and 12-months old mice, with 12-months old mice often defined as “middle-aged” and 20–30 months old animals as “old” (43, 45, 50). Whereas it might be possible to detect further differences in inflammatory capacity and ECM production at a later age, our data clearly reveal sex-dependent differences at both examined ages. This accentuates the importance of biological sex in inflammation and fibrosis processes and opens the avenue to perform further investigations in 20–30 months old mice on tissue and cellular level.

## 5. Conclusion

Our data indicate sex- and age-dependent differences in the inflammatory capability of cFB, which are related to variations in the cardiosplenic axis and cardiac fibrosis seen between female and male mice.

## Data Availability

The original contributions presented in the study are included in the article/**Supplementary Material**, further inquiries can be directed to the corresponding author/s.
