# Supplementary material for: Sex- and age-related differences in the inflammatory properties of cardiac fibroblasts: impact on the cardiosplenic axis and cardiac fibrosis
Source: Front Cardiovasc Med. 2023 Nov 20;10:1117419. doi: 10.3389/fcvm.2023.1117419 (PMC10694208; doi:10.3389/fcvm.2023.1117419)
Supplement: Supplementary file 1 [file Datasheet1.docx]

Supplement: Sex- and age-related differences in the inflammatory properties of cardiac fibroblasts: impact on the cardiosplenic axis and cardiac fibrosis

Kathleen Pappritz^1,2,3^, Sarah-Lena Puhl^4,5^, Isabel Matz^1,2,3^, Erik Brauer^6^, Yi Xuan Shia^5^, Muhammad El-Shafeey^1,2,3,7^, Suzanne E. Koch^2,8^, Kapka Miteva^2,9^, Christin Mucha^2^, Georg N. Duda^1,2,6^, Ansgar Petersen^1,2,6^, Sabine Steffens^5,10^, Carsten Tschöpe^1,2,3,11,12^, Sophie Van Linthout^1,2,3*^

^1^Berlin Institute of Health at Charité - Universitätsmedizin Berlin, BIH Center for Regenerative Therapies (BCRT), Berlin, Germany

^2^Berlin-Brandenburg Center for Regenerative Therapies, Charité – Universitätsmedizin Berlin, Campus Virchow Klinikum (CVK), Berlin, Germany

^3^German Center for Cardiovascular Research (DZHK), Partner site Berlin, Berlin, Germany

^4^Comprehensive Heart Failure Center, Universitätsklinikum Würzburg, Würzburg, Germany

^5^ Institute for Cardiovascular Prevention (IPEK), Ludwig-Maximilians-Universität (LMU) Munich, Munich, Germany

^6^Berlin Institute of Health at Charité – Universitätsmedizin Berlin, Julius Wolff Institute, Berlin, Germany

^7^Medical Biotechnology Research Department, Genetic Engineering and Biotechnology Research Institute (GEBRI), City of Scientific Research and Technological Applications, Alexandria, Egypt

^8^Department of Biomedical Engineering, Eindhoven University of Technology, Eindhoven, The Netherlands

^9^Division of Cardiology, Foundation for Medical Research, Department of Medicine Specialized Medicine, Faculty of Medicine, University of Geneva, Geneva, Switzerland

^10^German Center for Cardiovascular Research (DZHK), Partner Site Munich Heart Alliance (MHA), Munich, Germany

^11^Department Cardiology, Angiology, and Intensive Medicine (CVK) at the German Heart Center of the Charite (DHZC), Charité - Universitätsmedizin Berlin, Berlin, Germany

^12^Institute of Heart Diseases, Wroclaw Medical University, Wroclaw, Poland

*** Correspondence:**Sophie Van Linthout, PhD

Experimental Immunocardiology, Berlin Institute of Health at Charité - Universitätmedizin Berlin, BIH Center for Regenerative Therapies (BCRT)

Föhrer Strasse 15, 13353 Berlin, Germany

e-mail: [sophie.van-linthout@charite.de](mailto:sophie.van-linthout@charite.de), Phone: +49-(0)30-450539486, Fax: +49-(0)30-450539409

**
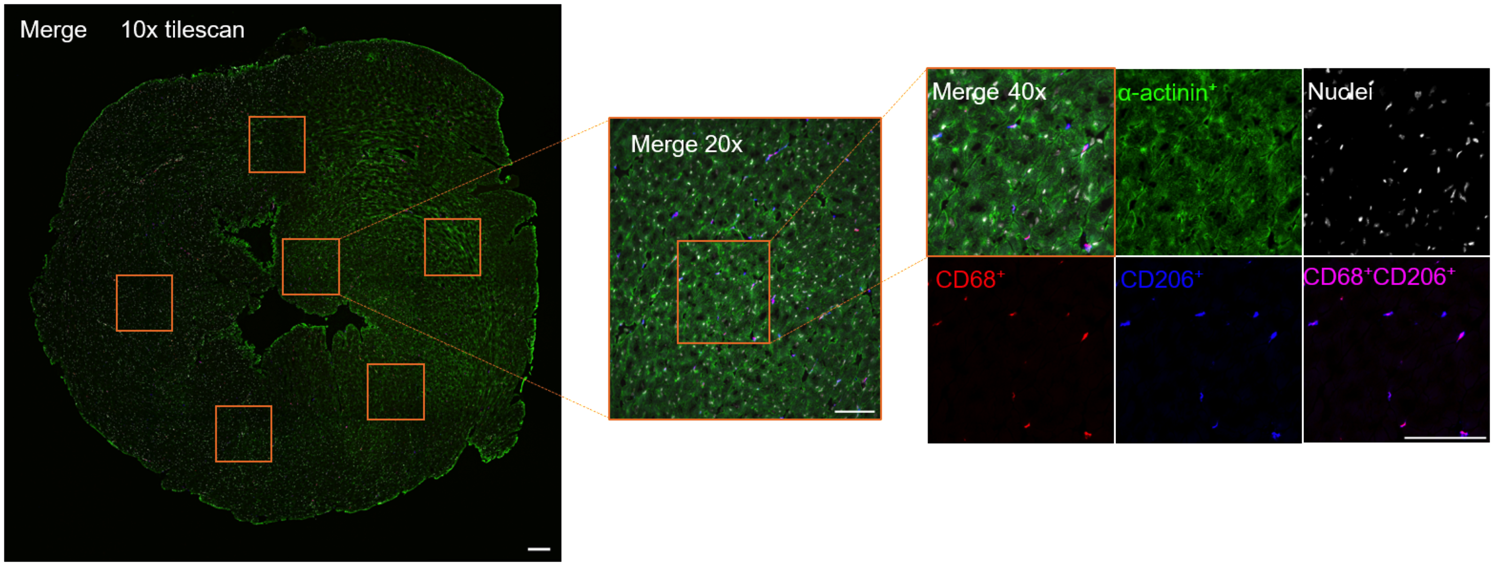
**

**Supplemental Figure 1. Scheme of the triple immunofluorescence staining of left ventricular tissue slides.** After the respective stainings, macrophage populations were manually counted via Image J in 2 non-consecutive sections per heart in 4-5 fields of view (FoV) per section, acquired at 20x magnification, and averaged per mouse. CD68^+^CD206^+^ and CD68^+^CD206^-^ subpopulations were quantified as percentage (%) of CD68^+^ cells.

**
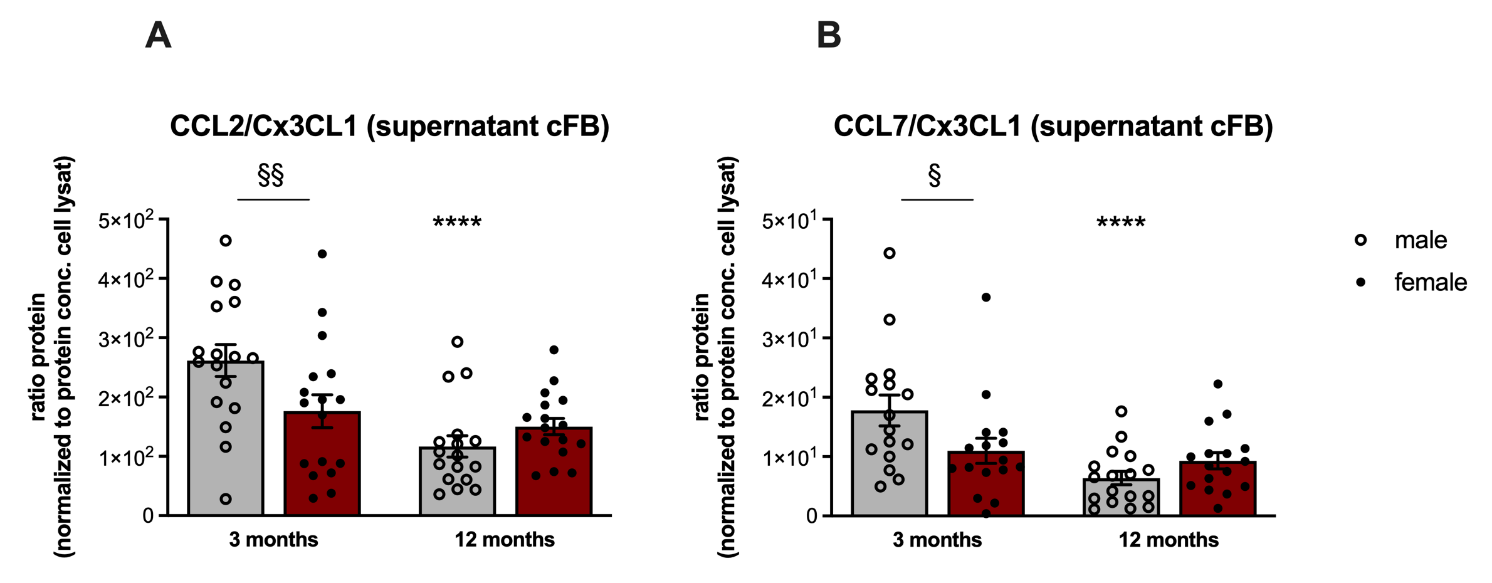
**

**Supplemental Figure 2. Sex- and age-related changes in chemokine protein concentrations of cardiac fibroblasts.** Analysis of the CCL2, CCL7, and Cx3CL1 protein concentration in cFB-derived supernatant via ELISA with subsequent normalization against the protein concentration of the cell lysate. Protein concentration of the cell lysate was determined via BCA method. Analogue to the unnormalized data, CCL2/Cx3CL1 (**A**) and CCL7/Cx3CL1 (**B**) ratios were calculated as parameters for monocyte attraction. Data are depicted as scatter plots with bars (male mice: gray bars; female mice: wine red bars), showing individual data points and the corresponding mean±SEM. For statistical analysis, Two-way ANOVA with Fisher´s LSD *post hoc* test was performed (^§^p<0.05, ^§§^p<0.01, ^§§§^p<0.001, ^§§§§^p<0.0001 male versus female mice; *p<0.05, **p<0.01, ***p<0.001, ****p<0.0001 versus 3-months old male mice; ^#^p<0.05, ^##^p<0.01, ^###^p<0.001, ^####^p<0.0001 versus 3-months old female mice; with n=5-6/group and N=3).


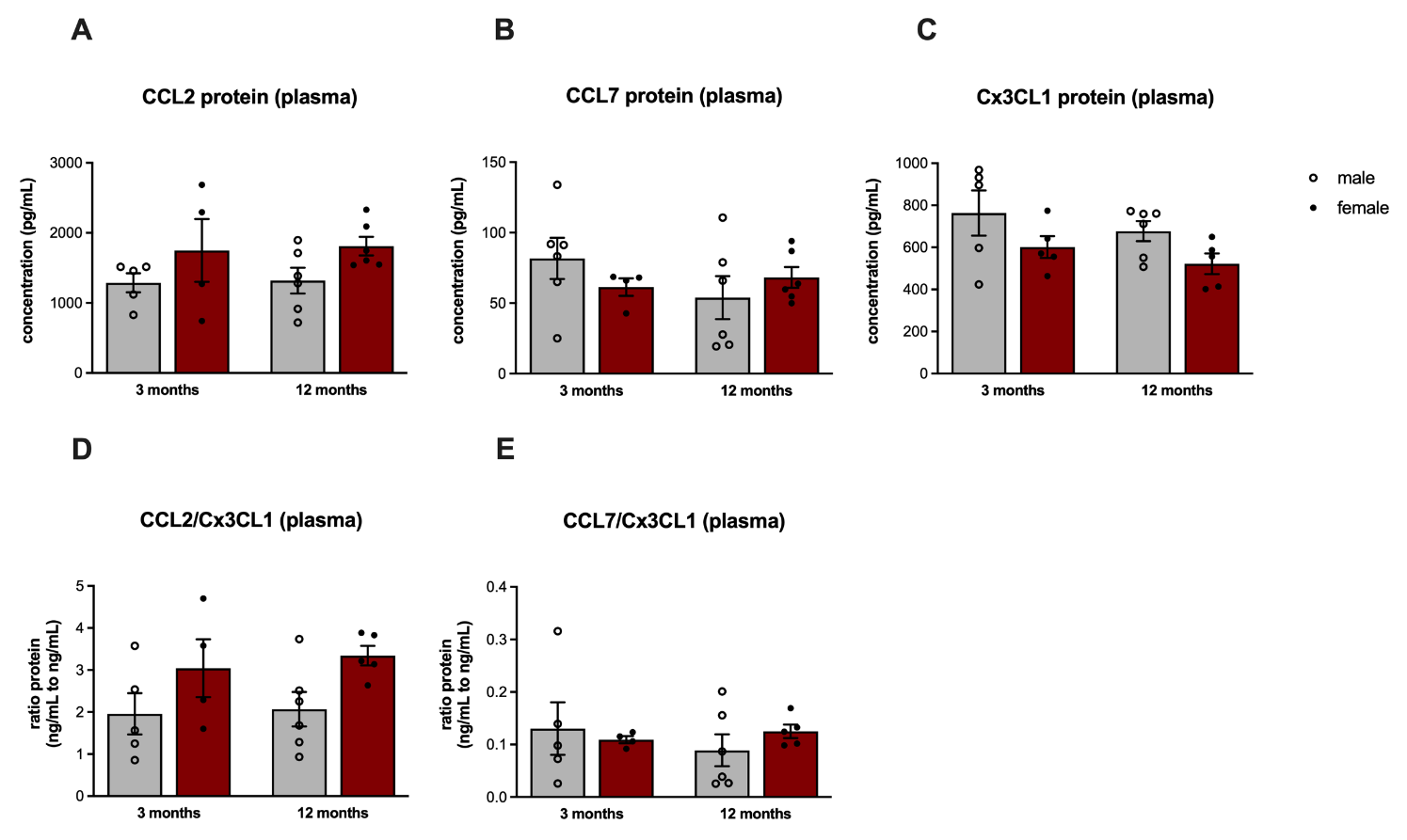


**Supplemental Figure 3. Sex- and age-dependent expression of pro- and anti-inflammatory chemokines in the plasma of male and female mice.** In order to investigate the circulating levels of CCL2 (**A**), CCL7 (**B**), and Cx3CL1 (**C**), plasma of male and female mice was analyzed. Similar to the analysis of the cell culture supernatant, protein concentrations were calculated separately as well as CCL2/Cx3CL1 (**D**) and CCL7/Cx3CL1 (**E**) ratios. Data are depicted as scatter plots with bars (male mice: gray bars; female mice: wine red bars), showing individual data points and the corresponding mean±SEM. For statistical analysis, Two-way ANOVA with Fisher´s LSD *post hoc* test was performed (^§^p<0.05, ^§§^p<0.01, ^§§§^p<0.001, ^§§§§^p<0.0001 male versus female mice; *p<0.05, **p<0.01, ***p<0.001, ****p<0.0001 versus 3-months old male mice; ^#^p<0.05, ^##^p<0.01, ^###^p<0.001, ^####^p<0.0001 versus 3-months old female mice; with n=5 for 3-months old male mice, n=4 for 3-months old female mice, n=6 for 12-months old male mice, n=6 for 12-months old female mice).
